# Supplementary material for: A self-supervised deep learning method for data-efficient training in genomics
Source: Commun Biol. 2023 Sep 11;6:928. doi: 10.1038/s42003-023-05310-2 (PMC10495322; doi:10.1038/s42003-023-05310-2)
Supplement: Supplementary file 2 — Supplementary Information [file 42003_2023_5310_MOESM2_ESM.pdf]

Supplementary Information

Supplementary Results

Comparison of Self-GenomeNet to baselines

The self-supervised baselines and our method are trained without using any labels (**Supplementary Fig. 1, Supplementary Fig. 2**). These models are then used for the downstream tasks. In the "supervised" baseline, there is no self-supervised pre-training, and randomly initialized weights are trained for downstream tasks.

Supplementary Figures

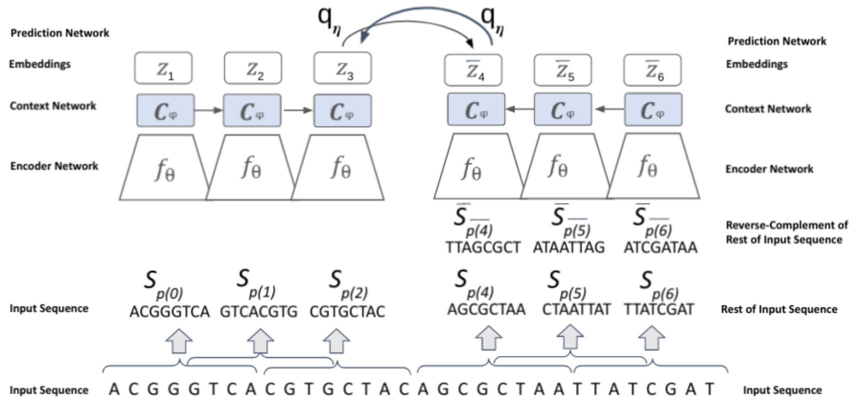

Supplementary Figure 1: Self-GenomeNet. The prediction network is used to predict representations of the reverse-complement of the neighboring sequence. The predicted sequences are of different lengths in *Self-GenomeNet*. A contrastive loss is used, which means that the matching sequence is predicted among other non-matching sequences in the batch.

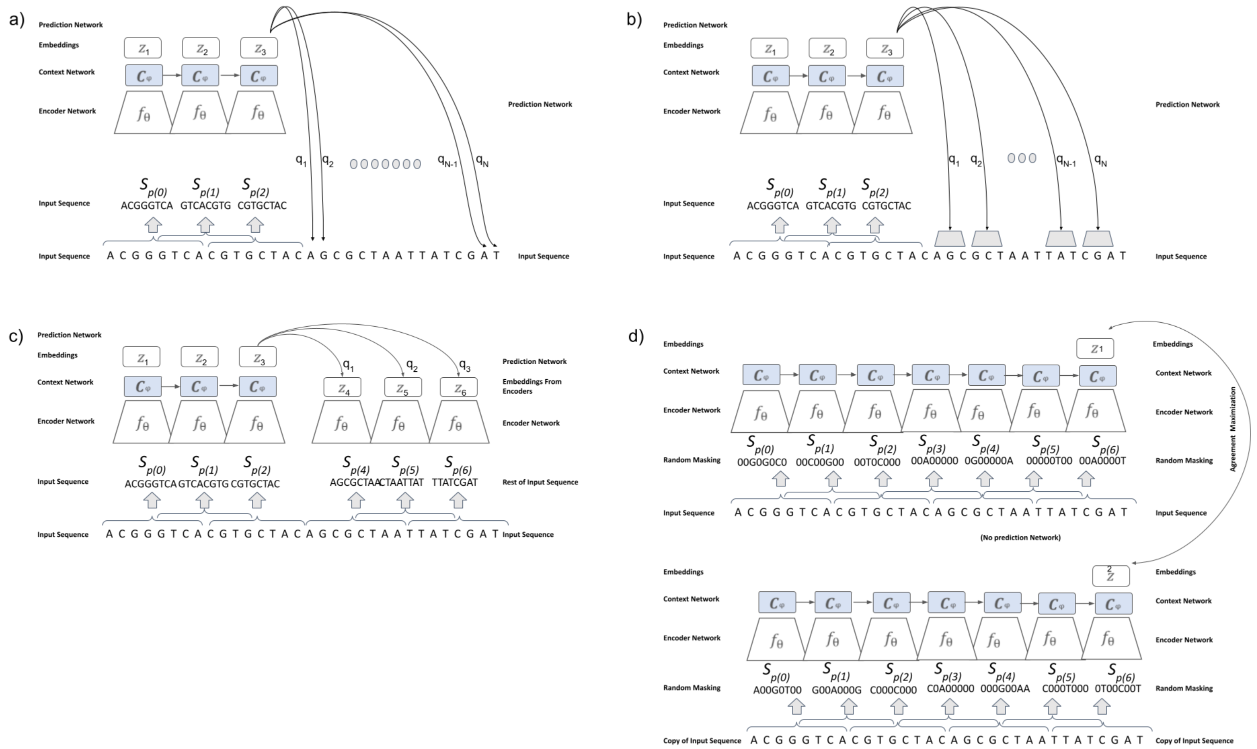

Supplementary Figure 2: Self-supervised baselines. a) Language Model - single nucleotide prediction (denoted as LM - single nt)[1], where each nucleotide is one-hot encoded separately as a vector of 4 and predicted separately using cross-entropy loss. b) Language Model - 3-gram prediction (LM - 3-gram), where each 3-gram (a group of 3 nucleotides) is one-hot encoded separately as a vector of 64 and predicted separately using cross-entropy loss. c) In Contrastive Predictive Coding[2,3], the representations of the short and non-changing length neighboring sequences are predicted (in the figure below, these sequences contain 8 nucleotides) using contrastive loss, which means that the matching sequence is predicted among other non-matching sequences in the batch. The representations of the predicted sequences are obtained by feeding them into the encoder network. d) Contrastive-sc[4] takes a sequence and a copy of the sequence and randomly masks the input sequence. Then, the agreement between the embeddings of two masked copies is maximized using contrastive loss, which means that the copied sequences predict each other among other mismatching copied sequences in the batch.

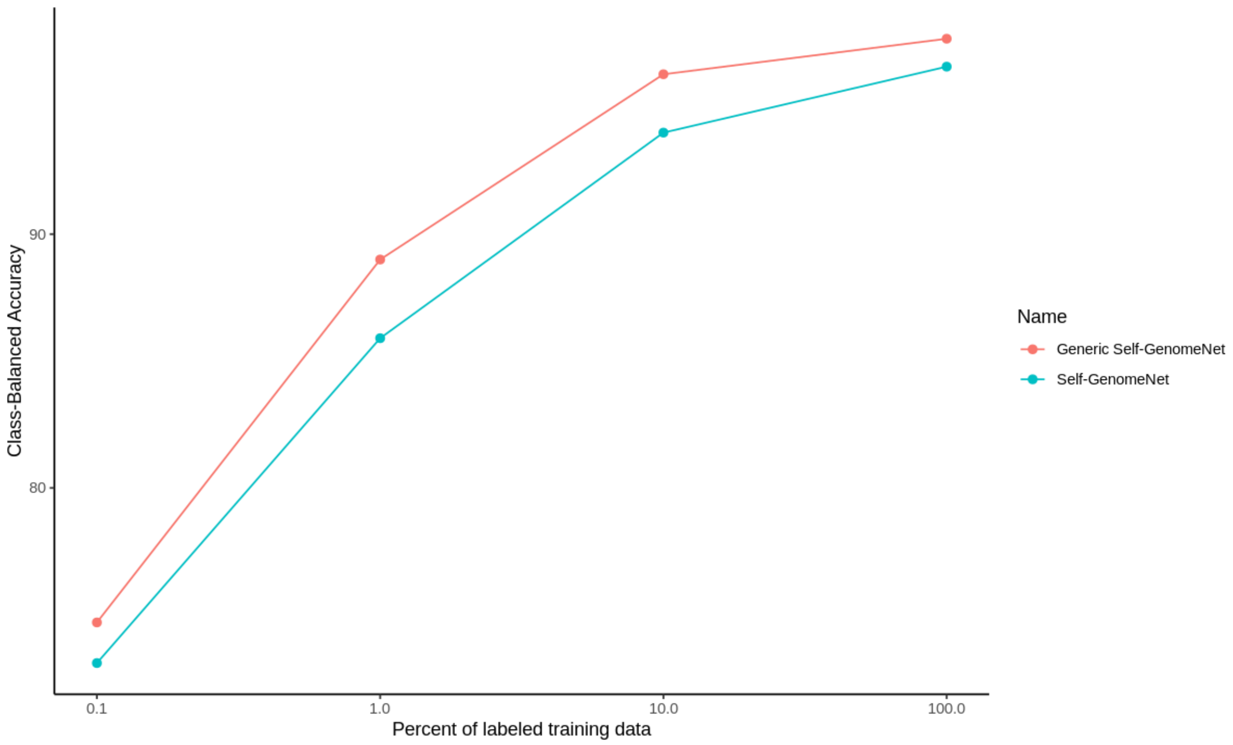

Supplementary Figure 3: Data-scarce settings performance comparison on the virus dataset. Generic Self-GenomeNet and Self-GenomeNet trained only on this dataset are evaluated. The generic *Self-GenomeNet*, which incorporates pre-training on virus, bacteria, and human data consistently outperformed the model pre-trained solely on virus data (the whole dataset) across all proportions of labeled data-scarce settings. We train the models without using labels and then successively withhold labeled samples of the viral dataset for 1,000 nt sequences to mimic scenarios where labels are scarce (from 100% of available labeled samples to 0.1%). Each point in the plots is trained separately using the corresponding amount of labeled data. The weights of the context and encoder models are initialized with the training results from the SSL task, but they are trained further (fine-tuned), together with the linear layer, on the new supervised task.

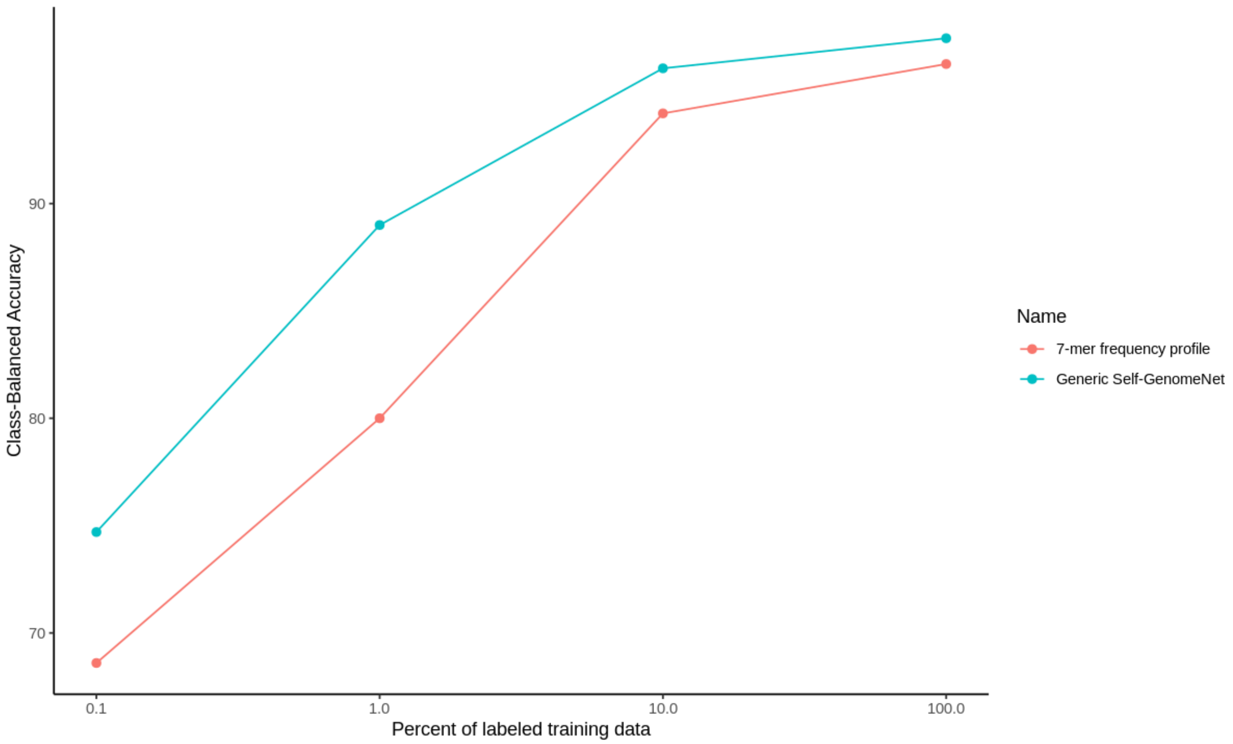

Supplementary Figure 4: Data-scarce Settings Performances of generic Self-GenomeNet and a supervised model that takes 7-mer profile as input. The generic *Self-GenomeNet* outperforms the CNN model proposed by Fiannaca et al[5]. *The input of this model is a 7-mer frequency - the normalized frequency of 7-mers observed in the sequence. While this model requires an additional pre-processing step (in order to create the histogram based on 7-mers) and has approximately six times the number of parameters compared to our Self-GenomeNet model, our approach consistently outperforms this baseline*, particularly in data-scarce settings. We train the generic Self-GenomeNet using virus, bacteria, and human data without using labels and then successively withhold labeled samples of the viral dataset for 1,000 nt sequences to mimic scenarios where labels are scarce (from 100% of available labeled samples to 0.1%). Each point of the generic Self-GenomeNet in the plots is trained separately using the corresponding amount of labeled data. The weights of the context and encoder models are initialized with the training results from the SSL task, but they are trained further (fine-tuned), together with the linear layer, on the new supervised task. The label “7-mer frequency profile” corresponds to the setting where we used the CNN model proposed by Fiannaca et al, where the weights are initialized randomly for the supervised task.

Supplementary References

1. Dai, A. M. & Le, Q. V. Semi-supervised sequence learning. *Adv. Neural Inf. Process. Syst.* **28**, (2015).
2. Lu, A. X., Zhang, H., Ghassemi, M. & Moses, A. Self-Supervised Contrastive Learning of Protein Representations By Mutual Information Maximization. *bioRxiv* 2020.09.04.283929 (2020) doi:10.1101/2020.09.04.283929.
3. van den Oord, A., Li, Y. & Vinyals, O. Representation Learning with Contrastive Predictive Coding. *arXiv [cs.LG]* (2018).
4. Ciortan, M. & Defrance, M. Contrastive self-supervised clustering of scRNA-seq data. *BMC Bioinformatics* **22**, 280 (2021).

5. Fiannaca, A. *et al.* Deep learning models for bacteria taxonomic classification of metagenomic data. *BMC Bioinformatics* **19**, 198 (2018).
