# Supplementary material for: A self-supervised deep learning method for data-efficient training in genomics
Source: Commun Biol. 2023 Sep 11;6:928. doi: 10.1038/s42003-023-05310-2 (PMC10495322; doi:10.1038/s42003-023-05310-2)
Supplement: Supplementary file 3 — Description of Supplementary Materials [file 42003_2023_5310_MOESM3_ESM.docx]

**Description of Additional Supplementary Files**

**File name:** Supplementary Data 1

**Description:** Source data for Figures 2, 3, 4 and Supplementary Figures 3 and 4.
